# Deodorized Garlic Decreases Oxidative Stress Caused by Lipopolysaccharide in Rat Heart through Hydrogen Sulfide: Preliminary Findings

**DOI:** 10.3390/ijms232012529

**Published:** 2022-10-19

**Authors:** Israel Pérez-Torres, Linaloe Manzano-Pech, Verónica Guarner-Lans, María Elena Soto, Vicente Castrejón-Téllez, Ricardo Márquez-Velasco, Álvaro Vargas-González, Raúl Martínez-Memije, Leonardo Del Valle-Mondragón, Julieta Anabell Díaz-Juárez, María Sánchez-Aguilar, Juan Carlos Torres-Narváez

**Affiliations:** 1 Department of Cardiovascular Biomedicine, Instituto Nacional de Cardiología Ignacio Chávez, Juan Badiano 1, Sección XVI, Tlalpan, Ciudad de México 14080, Mexico; 2 Department de and Hysiology, Instituto Nacional de Cardiología Ignacio Chávez, Juan Badiano 1, Sección XVI, Tlalpan, Ciudad de México 14080, Mexico; 3 Department de Inmunology, Instituto Nacional de Cardiología Ignacio Chávez, Juan Badiano 1, Sección XVI, Tlalpan, México City 14080, Mexico; 4 Department de Pharmacology, Instituto Nacional de Cardiología Ignacio Chávez, Juan Badiano 1, Sección XVI, Tlalpan, Ciudad de México 14080, Mexico; 5 Department de Electromechanical Instrumentation, Instituto Ncional de Cardiología Ignacio Chávez, Ciudad de México 14080, Mexico

**Keywords:** deodorized garlic, lipopolysaccharide, oxidative stress, heart failure, hydrogen sulfide

## Abstract

Deodorized garlic (DG) may favor the activity of the antioxidant enzymes and promote the synthesis of hydrogen sulfide (H_2_S). The objective was to test if DG favors an increase in H_2_S and if it decreases the oxidative stress caused by lipopolysaccharide (LPS) in rat hearts. A total of 24 rats were divided into 4 groups: Group 1 control (C), Group 2 LPS, Group 3 DG, and Group 4 LPS plus DG. The cardiac mechanical performance (CMP), coronary vascular resistance (CVR), and oxidative stress markers, such as total antioxidant capacity (TAC), glutathione (GSH), selenium (Se), lipid peroxidation (LPO), thiols, hydrogen sulfide (H_2_S), and the activities and expressions of thioredoxin reductase (TrxR), glutathione peroxidase (GPx), and glutathione-S-transferase (GST), cystathionine synthetase (CBS), cystathionine γ-lyase (CTH), iNOS, and eNOS-p, were analyzed in the heart. Infarct zones in the cardiac tissue were present (*p* = 0.01). The CMP and CVR decreased and increased (*p* ≤ 0.05), TAC, GSH, H_2_S, NO, thiols, and GST activity (*p* ≤ 0.01) decreased, and LPO and iNOS increased (*p* ≤ 0.05). The activities and expressions of TrxR, GPx, eNOS-*p*, CTH, and CBS (*p* ≤ 0.05) decreased with the LPS treatment; however, DG normalized this effect. DG treatment decreases heart damage caused by LPS through the cross-talk between the H_2_S and NO systems.

## 1. Introduction

Garlic (*Allium Savitum*) has been consumed by humans for thousands of years as part of the diet and it has been shown that it has beneficial effects on much pathology including dyslipidemia, atherosclerosis, platelet aggregation, hypertension, and cardiovascular disease (CVD), among others [1]. Different studies in both animal and human models with cardiovascular damage have shown that garlic reduces damage in this organ without causing collateral effects [1]. The beneficial effect of different presentations of garlic (raw, aged, or powdered) has been attributed to active metabolites, such as E/Z-ajoene, S-allyl-cysteine (SAC), diallyl thiosulfonate (allicin), S-allyl-cysteine sulfoxide (alliin), gallic acid, rutin, protocatechuic acid, quercetin, S-allylmercaptocysteine (SAMC), diallyl sulfide (DAS), diallyl trisulfide (DATS), and diallyl disulfide (DADS), among others. These garlic compounds are soluble in water, alcohol, and oil and they are responsible for antioxidant activity through the activation of the nuclear factor erythroid 2-related factor 2 (Nrf2) pathway [2]. Sulfide components are also present in high amounts and they increase the activity of enzymes that play an important role in the synthesis of hydrogen sulfide (H_2_S) [2].

In normal physiological conditions, cystathionine β-synthetase (CBS), cystathionine γ-lyase (CTH or CSE), and 3-mercaptopyruvate transferase (3-MST) endogenously produce the gas H_2_S [3]. H_2_S penetrates cell membranes without needing transporters since it is a lipophilic molecule, and it then participates as a signaling messenger regulating important processes in the body. H_2_S binds to ion channels, such as K_ATP,_ Ca^2+^, Cl^−^, and it modulates the TRPV1 and TRPA1 receptors. The expression of antioxidant response elements (AREs), which is controlled by the Keap1-Nrf2 pathway, is also modulated by this gas [4]. The liberation of H_2_S from garlic compounds needs molecules, such as glutathione (GSH), thiol groups, cysteine, and other substances [5]. Organic polysulfides derived from garlic interact with GSH to generate H_2_S and after being transported through the cell membrane, it leads to hyperpolarization in red blood cells. In vascular smooth muscle cells, H_2_S causes relaxation [6]. Furthermore, GSH may be synthetized by a trans-sulfuration pathway that is regulated by CBS and CSE, which are essential enzymes for L-cysteine biosynthesis in the presence of vitamin B [7].

On the other hand, deodorized garlic (DG) results from the pulverization and denaturation of this vegetable, thus remaining without flavor or aroma but keeping the consistent benefits of raw garlic. It seldom has adverse reactions. DG stabilizes compounds with antioxidant properties, such as allicin, SAC, SAMC, DAS DATS, and DADS. These stable compounds exert antioxidant actions by eliminating ROS, and favor an increase in the enzymatic and non-enzymatic antioxidant systems [8].

In addition, sepsis is a condition caused by viruses, bacteria, and fungi, or by a combination. Studies in human patient and animal models with septic shock have shown that there is an increase in the reactive oxygen species (ROS) and depletion of the non-enzymatic and enzymatic antioxidant systems [9]. In this condition, this affects the proper functionality of different organs, such as the myocardium. In this sense, in severe sepsis caused by an inadequate infection response of the host, the cardiovascular system favors an elevation of the cardiac output and it drastically decreases peripheral resistance, thus leading to arterial dilatation that results in progressive hypotension and production of refractory catecholamines [10]. This may contribute to severe cardiovascular failure with the possibility of a fatal outcome [11]. In addition, excessive decreases in peripheral resistance or its prolongation in time may contribute not only to ROS generation but also to the release of pro-inflammatory cytokines. For example, in cardiomyocytes treated with lipopolysaccharide (LPS) IL-1β and IFN-α, there is an increase in the peroxynitrite (ONOO^−^) levels that is associated with the overexpression of inducible nitric oxide synthase (iNOS) and deleterious effects in the myocardial energy balance. It attenuates the myocardial inotropic response to β-adrenergic stimulation and induction of necrosis [12]. Therefore, the objective of this study was to demonstrate if DG favors the increase in the H_2_S and decreases the oxidative stress (OS) in the heart after a period of ischemia and reperfusion (I/R) exposed to LPS insult.

## 2. Results

### 2.1. Cardiac Mechanical Performance (CMP)

**Figure 1:**
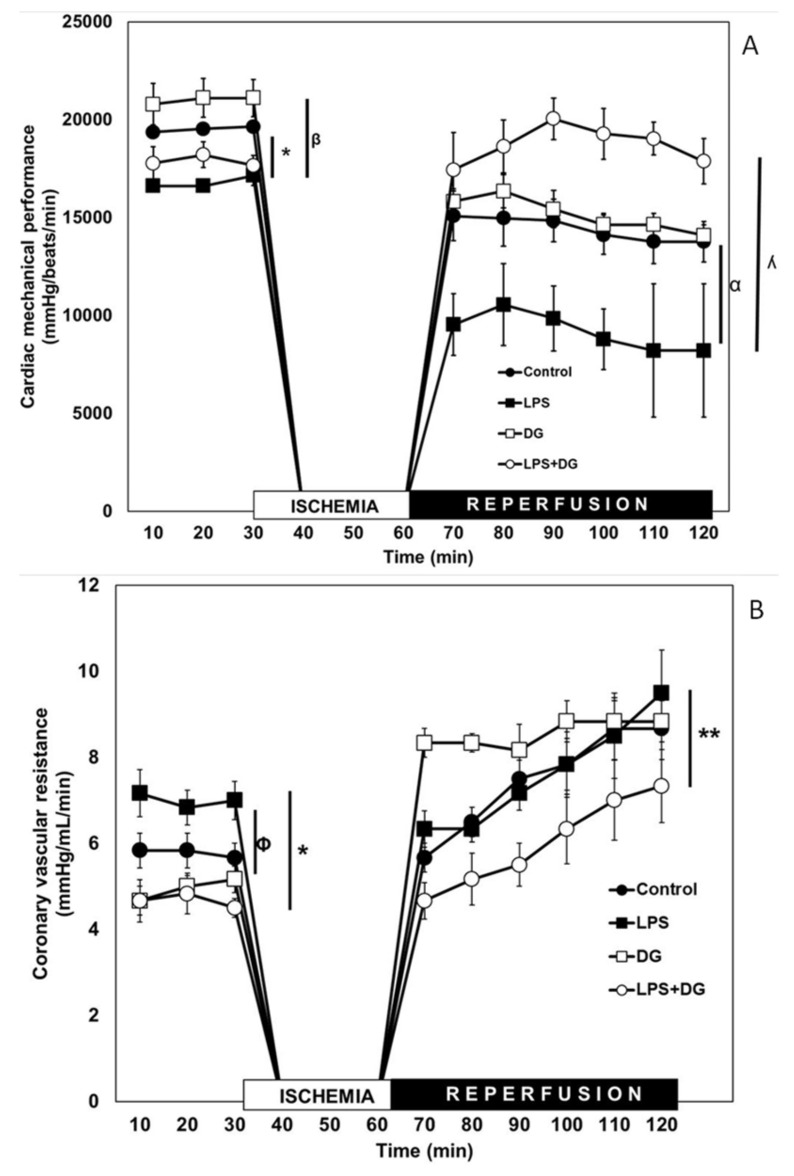
(**A**) Cardiac mechanical performance and (**B**) coronary vascular resistance of hearts with ischemia and reperfusion from the C (black circles), LPS (black squares), DG (white squares), and LPS + DG (white circles) groups. The hearts were isolated and dissected from rats with different treatments and perfused for 30 min. After that time, an ischemia-reperfusion period (I/R) was applied, I = 30 min and R = 60 min. Each heart was its own control to compare the pre-ischemic period vs. I/R, n = 6, *p* ≤ 0.05. CMP = Pre-ischemic period significance. * Control vs. LPS *p* = 0.001, ^β^ LPS vs. DG *p* = 0.001. Reperfusion significance: ^α^ DG vs. LPS + DG *p* = 0.02, ^ƛ^ LPS vs. LPS + DG *p* = 0.03. CVR = Pre-ischemic period: ^Ø^ LPS vs. DG *p* = 0.003. * LPS vs. LPS + DG *p* = 0.002. Reperfusion significance: ** DG vs. LPS + DG *p* ≤ 0.05.

Figure 1A shows the results obtained for the CMP in two experimental conditions: The pre-ischemic period and the I/R period (30 and 60 min, respectively). In the pre-ischemic period, the CMP decreased by 20% in the rats with LPS vs. C rats (*p* ≤ 0.05). In the group treated only with DG, no significant changes were observed for any of the groups. In the rats treated with LPS + DG, the mechanical activity of the heart was restored with respect to the LPS group (*p* = 0.01). We observed that, in this period, the effect of the decrease in CMP caused by LPS was eliminated by DG in the LPS + DG group. In the same figure, it can be observed that, during the I/R period, the CMP of the hearts of the C rats decreased by, on average, 26% compared to the C group (before I/R). The LPS group decreased by 45% when compared to the same group in the pre-ischemic period. DG treatment decreased by 38% when compared to the same group and it did not prevent the I/R damage. This means that the hearts treated with LPS had 15% more damage than the C group (45–26 = 15). The damage caused by I/R in the CMP with the treatment with LPS was prevented in the LPS + DG group (*p* = 0.001). It is important to highlight that the I/R damage (decrease in CMP) was also maintained from the beginning to the end of reperfusion. No significant differences were found between groups C vs. DG, but in the LPS group, an important tendency of the I/R damage to increase was observed. It is important to highlight that treatment with DG in the LPS + DG group prevented I/R damage when compared to the LPS group.

### 2.2. Coronary Vascular Resistance (CVR)

During the pre-ischemic period, CVR increased in the LPS group in comparison with C and LPS + DG groups (*p* ≤ 0.05). In all groups, I/R damage manifested by an increase in CVR as a function of time and with respect to their own controls. It is noteworthy that, when comparing the LPS group vs. the LPS + DG group, CVR decreased on average from 8 to 6 mmHg/mL/min (Figure 1B).

### 2.3. Histological Analysis

**Figure 2:**
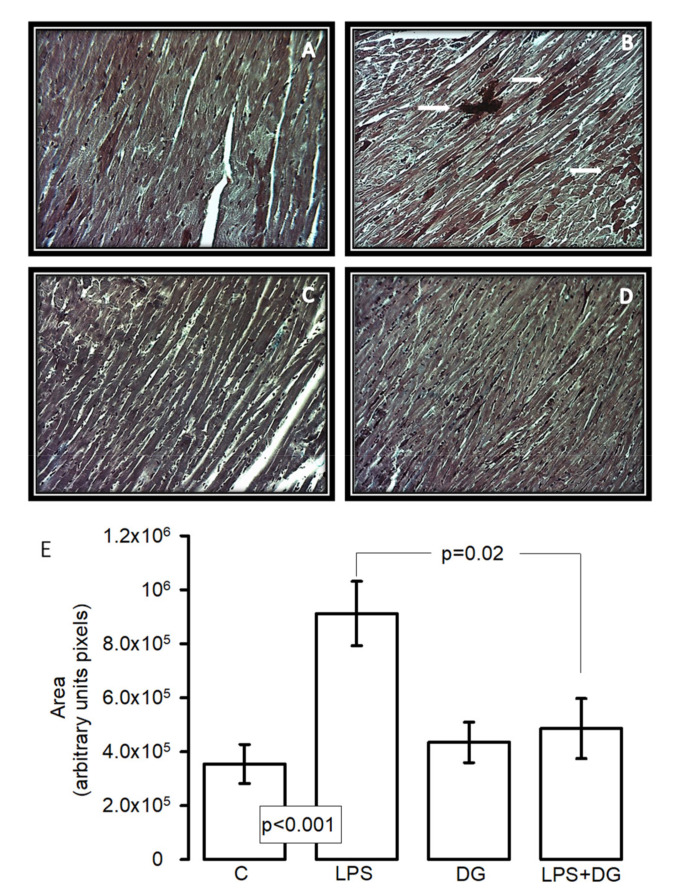
Representative photomicrographs to 25× of heart tissue after perfusion (30 min), ischemia (30 min), and reperfusion (60 min) in experimental groups. (**A**) Control, (**B**) LPS, (**C**) DG, (**D**) LPS + DG, and (**E**) down-panel histogram that represents the densitometric analysis of the size of the infarct zones between the cardiac fibers. The arrows indicate the area of infarction in the experimental group. Values are the mean ± SE (n = 6). The tissue was processed according to conventional histological procedures, and histological sections were made and stained by Masson’s trichrome stain at 25×. Abbreviations: C = control, DG = deodorized garlic, LPS = lipopolysaccharide.

Figure 2A shows representative microphotographs of the C, LPS, DG, and LPS + DG groups. The images were obtained from areas irrigated by the left anterior descending coronary artery and were taken from the tip of the heart and from the anterior wall of the left ventricle, at approximately two-thirds of the anterior to the ventricular septum. No alterations were found in the histological sections of the cardiac tissue in the C and DG groups. Compact bundles of the contraction bands in the myocytes were separated by fibrous bands. Intercalated disks are focally distinguished. Ovoid nuclei in the myocytes were observed as in the normal cardiac muscle. However, damage was observed in the cardiac tissue with the LPS treatment; infarct zones were observed. The damage was prevented in the hearts in the groups treated with LPS + DG. The densitometric analysis in the histological samples demonstrated that the treatment with LPS significantly increased the size of the infarct zones in comparison with the C and LPS + DG groups (*p* < 0.001 and *p* = 0.02, respectively, Figure 2B).

### 2.4. Oxidative Stress Markers

Table 1 shows that the TAC and GST activity significantly decreased in the LPS group in comparison with C and LPS + DG groups (*p* ≤ 0.01). The GSH levels and thiols showed a similar tendency in the same groups but with more significant change (*p* ≤ 0.001). However, the LPO level increased in the LPS group vs. the C and LPS + DG groups (*p* ≤ 0.001) and the Se concentration increase in the LPS + DG group in comparison with the LPS group (*p* ≤ 0.001).

### 2.5. Enzymatic Activities and Expressions of the TxrR and GPx

**Figure 3:**
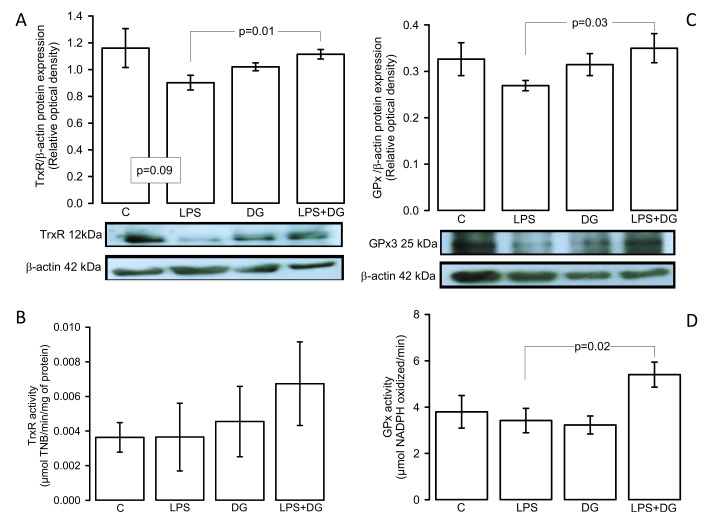
Enzymatic activities and expressions of the (**A**) TxrR expression, (**B**) TrxR activity, (**C**) GPx expression, and (**D**) GPx activity. Values are expressed as mean ± SE (n = 6). Abbreviations: C = control, DG = deodorized garlic, LPS = lipopolysaccharide, TrxR = thioredoxin reductase, GPx = glutathione reductase.

Figure 3A shows that the TrxR expression decreased in the LPS group in comparison with the LPS + DG group (*p* = 0.01). However, the activity showed a tendency to increase without reaching a significant change (Figure 3B). On the other hand, the GPx expression and activity showed an increase in the LPS + DG group vs. the LPS group (*p* = 0.03 and *p* = 0.02, respectively (Figure 3C,D), and tendency to decrease in the LPS group vs. the C group without showing significant changes (*p* = 0.06).

### 2.6. Expressions of the CTH, CBS Enzymes and H_2_S Concentration

**Figure 4:**
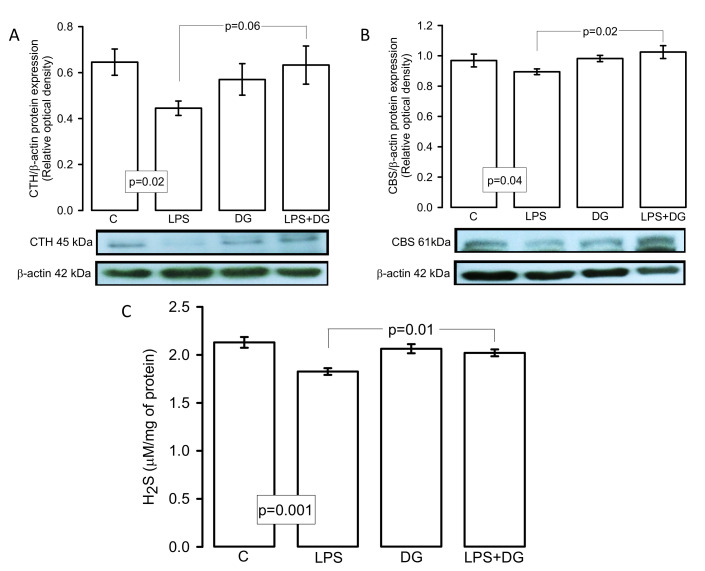
Expressions of the (**A**) CTH expression, (**B**) CBS expression, and (**C**) H_2_S concentration. Values are expressed as mean ± SE (n = 6). Abbreviations: C = control, DG = deodorized garlic, LPS = lipopolysaccharide, CBS = cystathionine synthetase, CTH = cystathionine γ-lyase, H_2_S = hydrogen sulfide.

Figure 4A,B show the CTH and CBS expressions in the heart homogenate, where a significant decrease was present in the C group vs. the LPS group (*p* = 0.02 and *p* = 0.04, respectively). However, only the CBS expression presented a significant increase in LPS + DG vs. LPS (*p* = 0.02). There was only a tendency to increase in the CTH without reaching a statistical change (*p* = 0.06). With respect to the concentration of H_2_S, there was a decrease in the C and LPS + DG groups in comparison to the LPS group (*p* = 0.001 and *p* = 0.01, respectively, Figure 4C).

### 2.7. Expressions of the iNOS and eNOS Enzymes and eNOS-p Activity

**Figure 5:**
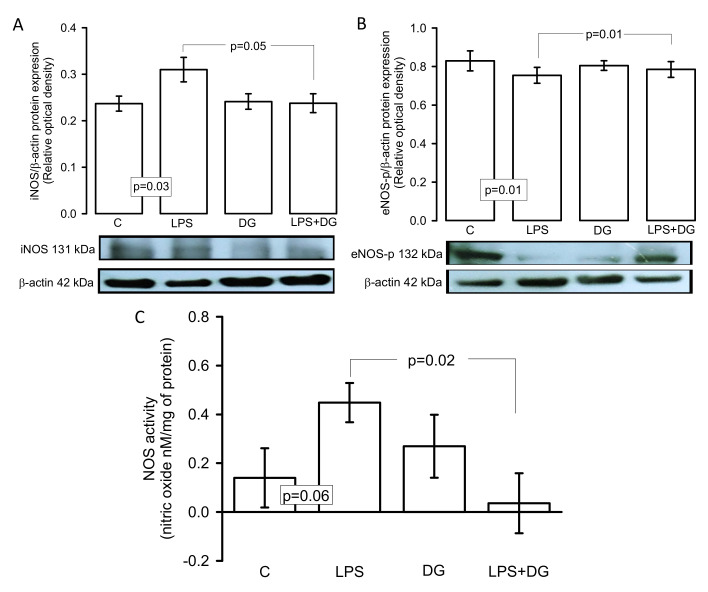
Expressions of the (**A**) iNOS-p expression, (**B**) iNOS expression, and (**C**) NO concentration. Values are expressed as mean ± SE (n = 6). The NOS activity was quantified using a Clark-type electrode attached to an oximeter and values are expressed as concentration of NO nM/mg of protein. Abbreviations: C = control, DG = deodorized garlic, LPS = lipopolysaccharide, eNOS-p = endothelial oxide nitric synthase phosphorylated, iNOS = inducible oxide nitric synthase, NO = nitric oxide.

Figure 5A,B show that the iNOS and eNOS-p expressions in the heart homogenate significantly increased and decreased (*p* = 0.03 and *p* = 0.01), respectively, in the C group in comparison to the LPS group. However, treatment with DG in the LPS group restored these changes (*p* = 0.05 and *p* = 0.01, respectively) in the iNOS and eNOS-p expression. In addition, the indirect activity of the NOS showed an increase in the NO production in the heart homogenate of the LPS group in comparison with the LPS + DG group, and a tendency in comparison with the C group without reaching a statistical change (*p* = 0.06, Figure 5C).

## 3. Discussion

It is well-established that LPS of *Escherichia Coli* may trigger a high inflammatory stimulus and lead to severe sepsis accompanied by loss of redox homeostasis with an increase in ROS, affecting the proper functionality of the myocardium. This may end in heart failure since complex changes occur in the heart, including mechanical, structural, biochemical, and electrical [13]. There may be presence of cardiac arrhythmias, coronary heart disease, left ventricular hypertrophy, and congestive heart failure [14].

However, garlic consumption in any of its different presentations may protect from cardiovascular diseases (CVD) [15]. Therefore, the goal of this study was to demonstrate if DG favors the increase in H_2_S and decreases OS in the heart after a period of I/R exposed to LPS insult. Our results show evidence of histological and physiological changes with LPS treatment. Myofibrils were altered, infarct zones were present, and there were changes in CMP and CVR observed through of the I/R. In this sense, the sepsis process may decrease mitochondrial function and this may contribute to a diminution of the ATP levels reaching the myofibrils, leading to a decrease in CMP [16]. Treatment with DG improved CMP and CVR. This suggests that DG could fight negative inotropic and chronotropic effects in the heart in sepsis [17] and this could be mediated by the sulfur components that are associated with H_2_S production [18]. Furthermore, within the toxic effects of LPS, myocardial dysfunction derived from a generalized inflammatory state known as systemic inflammatory response syndrome is present [19].

The consequences of this syndrome in the heart are alterations in coronary perfusion, myocyte hypertrophy, apoptosis, and interstitial fibrosis [20]. However, treatment with garlic could favor the decline of this syndrome. In this sense, in a rat model with metabolic syndrome, treatment with aged garlic extract decreased the CVR and this was associated with its antioxidant properties [17]. In another study, SAC had a dose-dependent protective effect against isoproterenol-induced cardiotoxicity in rats [21]. Therefore, our results suggest that treatment with DG could contribute to the prevention of changes in the MCP and CRV that are associated with cardiac function and that could contribute to restoring or decreasing the size of the infarct produced by the LPS treatment [22,23]. Another study showed that H_2_S may diminish heart injury by I/R and promote the activity of the adenosine triphosphate-sensitive potassium channels that are affected by several pro-inflammatory cytokines. It also reduces H_2_O_2_ [24].

**Figure 6:**
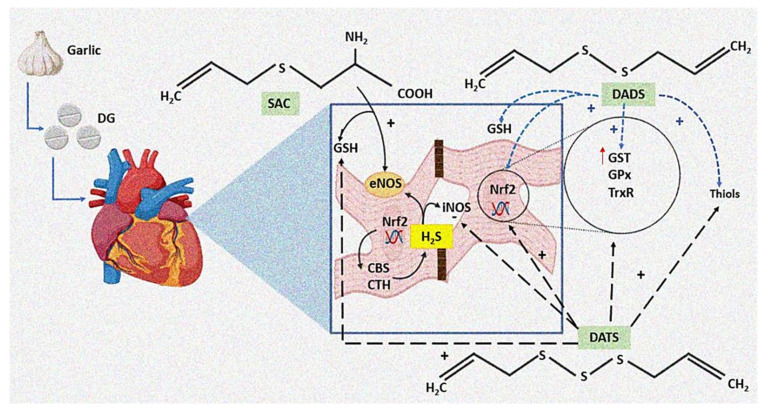
Mechanisms by which DG treatment may down-regulate the negative inotropic and chronotropic effects in the heart in sepsis. A possible mechanistic explanation for the beneficial effect of the DG treatment on the heart is through the H_2_S production. The active organic polysulfides, such as SAC, DADS, and DATS, which are provided by DG, act as H_2_S donors in the presence of thiols and GSH through an increase in the CBS and CTH activity. These polysulfides could also increase the expression of Nrf2 that modulates the overexpression of the antioxidant enzymes, such as eNOS, GST, GPx, and TrxR. Abbreviations: SAC = S-allyl-cysteine, DAS = diallyl sulfide, DATS = diallyl trisulfide, Nrf2 = nuclear factor erythroid 2-related factor 2, CBS = cystathionine β-synthetase, CTH = cystathionine γ-lyase, GSH = glutathione, iNOS = inducible nitric oxide synthase, H_2_S = hydrogen sulfide synthesis, eNOS = endothelial nitric oxide synthase, DG = deodorized garlic, TrxR = thioredoxin reductase, GPx = glutathione reductase, GST = glutathione-S-transferase.

A possible mechanistic explanation for the beneficial effect of treatment with DG on the heart is through the production of H2S since this gas is involved in multiple physiological functions and potentially contributes to pathological states, such as hypertension, stroke, vascular reactivity, cardiac failure, obesity, and diabetes [25]. Moreover, the effect of garlic has been primarily attributed to the active organic polysulfides that it contains (SAC, DADS, and DATS), which are donors of H2S in the presence of thiols and thiol-containing compounds, such as GSH [26]. Figure 6 describes this process in the cardiomyocytes.

In this sense, when the increase in the production of H_2_S caused by DATS was compared to that caused by sodium sulfide or sodium hydrosulfide, DATS was found to gradually increase its production over an extended period of time and to elevate endogenous H_2_S concentrations after myocardial I/R. It resulted in significant reductions in the areas of infarct and decreased circulating concentrations of markers of cardiac injury, including cardiac troponin I [27]. Accordingly, our results show that the DG treatment increased both GSH and thiols, which were decreased by LPS. This suggests that treatment with DG could favor H_2_S production. The DG treatment could also provide the essential amino acids needed for the de novo synthesis of GSH or for the restoration of GSH and thiol levels. A deregulation of GSH homeostasis compromises cardiomyocytes in heart failure and the oxidative damage caused may lead to cell death [28]. A possible explanation is that H_2_S increases the transport of cysteine, redistributing GSH into the mitochondria and protecting the cells from OS [28]. A study showed that treatment with aged garlic extract (AGE) in cultured endothelial cells prevented OS by increasing the cellular concentration of cysteine, thiols, and GSH. This suggests that AGE and DG could prevent intracellular GSH depletion and modulate the GSH redox cycle [29].

GSH is required to regulate the endogenous redox homeostasis in the heart. It also acts as a nucleophilic molecule and a reducing agent that reacts with and eliminates electrophilic or oxidizing species. Therefore, it prevents the damage of protein, lipids, nucleic acid, and other molecules. In conditions such as sepsis, the GSH and H_2_S [30] are depleted while trying to counteract OS [31]. However, the DG treatment may increase the CBS and CTH enzymes, which are needed for L-cysteine biosynthesis, thus providing the substrate that undergoes the trans-sulfuration pathway to produce GSH [32]. Many H_2_S-generating reactions are catalyzed by employing cysteine and homocysteine as substrate. Substrate availability is crucial for the regulation of this mechanism [28].

Our results show that both CBS and CTH enzymes increased their expression and that H_2_S levels were increase by the DG treatment. This suggest that the DG treatment may favorably impact the heart in a sepsis condition and that it could restore the redox homeostasis in part by the H_2_S, CBS, CTH, GSH, and thiol elevations in this organ. In this sense, treatment with DATS increased H_2_S levels and protected against ischemic damage in the hearts of mice with I/R injury through overexpression of CTH [33].

However, another possible mechanistic explanation of the cardio protection is that H_2_S increases could be mediated via cross-talk with nitric oxide (NO) [23]. Different studies have shown that the administration of NO donors enhances the H_2_S-producing enzymes CBS and CTH, promoting vessel relaxation via activation of the activity of the endothelial NO synthase (eNOS) [34]. Sulfur components of garlic by themselves can increase the eNOS activity and favor the production of NO, which is also diminished in sepsis [35]. Moreover, there are high levels of arginine in garlic powder, which may elevate the activity of eNOS in a dose-dependent manner. The activity of eNOS was increased through an elevation of the arginine content in macrophages stimulated with LPS [35]. Therefore, NO and H_2_S could act synergistically and play a critical role in modulating cardiomyocyte contractile functions. In sepsis, both molecules are depleted and treatment with DG may restore them, as shown by our results where there was an increase in the expression of eNOS-p [5]. However, the overproduction of ROS in sepsis is associated with inflammation and this condition triggers the up-expression of iNOS accompanied by a high production of NO, which reduces the intracellular Ca^2+^. It also reduces the Ca2þ spark frequency and depletes ATP in the cardiomyocytes. This results in a depression of the cardiovascular system, a lowering of blood pressure and cardiac output, and an attenuation of pressor responses to vasoactive agents [36,37]. Our results show an overexpression of iNOS associated with elevated NO production in LPS that was restored by treatment with DG.

Furthermore, among some garlic compounds that show anti-inflammatory effects are 2-linoleoyl glycerol pyruvate and 5-hydroxy methylfurfural. 2-linoleoyl glycerol pyruvate reduces bacterial LPS and suppresses the NO levels and pro-inflammatory cytokines by inhibition of mitogen-activated protein kinases signaling pathways. 5-hydroxy methylfurfural may diminish the adhesion of monocytes in human umbilical vein endothelial cells incubated with TNF-α through the suppression of vascular cell adhesion molecule-1 expression, NF-kB activation, and ROS generation [38]. Recent research has found that AGE components exert cardio protection attributed to its active ingredient S-allylcysteine that increases NO levels by stimulating eNOS expression but depletes the iNOS [39]. This suggests that DG treatment could improve the performance of the heart, via eNOS activation while decreasing the iNOS expression [40]. In this sense, a previous study demonstrated that SAC administration in rats significantly decreased the expression of NF-*κ*B, TNF-α, and iNOS [41].

On the other hand, garlic contains antioxidants, such as polyphenols and gallic acid, that support the protective mechanisms against oxidative damage by increasing antioxidant enzymes through of the regulation the Keap1-Nrf2 pathway [42]. An increase in H_2_S levels can stabilize Nrf2 by inhibiting the Keap1 protein, leading to the activation of several genes that encode for CBS, CTH, GPx, and TrxR [43]. However, other mechanisms have been proposed, such as the S-sulfhydration of Keap1, leading to its dissociation from Nrf2, its translocation to the nucleus, and the subsequent activation of the ARE genes that encode for proteins that are part of the enzymatic antioxidant system [44]. In this sense, our results show that the DG treatment increases the activities and expressions of GPx and TrxR in the heart homogenate from rats pretreated with LPS. In this regard, garlic contains selenium (Se), which might increase the activity of both enzymes, which can decrease the H_2_O_2_. TrxR also participates in the reduction in the number of thiols that are formed between the bridges of the proteins and enzymes [45]. Our results show that the Se was increased by the DG treatment. This result suggests that the DG treatment could act through two means: (a) modulating and favoring these enzymes through the H_2_S-Nrf2 axis on overexpression, and (b) by contributing to the elevation of the Se levels, forming part of the catalytic center of both enzymes, and favoring their activity [17]. Therefore, treatment with DG decreases the OS and increases the reduction between the disulfide bonds in the proteins of the microfibrils in the heart. In this sense, DATS may modulate the expression and activity of the Trx/TrxR system, which is also dependent on H_2_S production [46].

In addition, the results presented here suggest that the favorable changes on both enzymes and the increase in GSH levels, thiols, and Se could be reflected in the increase in TAC, which is the result of the enzymatic and non-enzymatic antioxidant systems and, as result of this, the LPO index decreased, thus favoring the reduction and impact of OS on the heart with I/R due to the treatment with LPS. In this regard, a study showed that doxorubicin in mice causes arrhythmia, ventricular extra systole, intraventricular blockade, and bradycardia, but that AGE prevented myocardial damage and decreased the LPO index [47]. However, a decrease in the LPO index, such as that shown our results, may also be due to the garlic properties that may favor GST activity. This enzyme of phase II is responsible for lipid-oxidized detoxification through the conjugation with GSH [48]; the decrease in the activity of this enzyme could favor the accumulation of the oxidized lipids by ROS in the cell bilipid membrane. Therefore, our results suggest that the DG treatment could favor the increase in the activity of this enzyme. Different compounds of garlic are linked to the increase in the activity, expression, or mRNA formation of this enzyme, including DAS, DADS, and DATS [49]

## 4. Materials and Methods

Twenty-four male Wistar rats of 300–350 g were divided into four groups with six animals per group. The groups were: Group 1 control (C), Group 2 LPS, Group 3 DG, and Group 4 LPS plus DG. The rats were provided by the Laboratory Animal Care Facility of the National Institute of Cardiology, “Ignacio Chávez”, in México. All procedures for the handling of animals were approved by the Institutional Ethics Committee and were in accordance with the National Rules for the care and handling of experimental animals (SAGARPA, NOM-062-ZOO-1999). Rats ate a standard diet (Lab diet 5008, PMI Nutrition International, Richmond, IN, USA), at libitum. The animals were placed in plastic boxes and were kept under 12 h light/obscurity cycles and environmental temperature ranging from 18 to 26 °C, for optimal conditions.

### 4.1. Deodorized Garlic

*Cursive Sativum* or Chinese garlic tablets (Ajolín Forte^®^ plus, Deodorized Garlic) of 500 mg were diluted in water and provided at libitum. The solution was changed every 12 h for 1 month. The nutritional information for the tablets showed a total of 600 μg of sodium, 750 mg fat, 20 g carbohydrates, and 0 g protein.

### 4.2. LPS Inoculation

LPS was provided by Creative-Biolabs vaccine; lipopolysaccharide (*E. coli* 0111:B4 strain) (VAdv-Ly0030) at a ratio of 15 mg/kg and diluted in saline was intraperitoneally applied to the rats in Groups 2 and 4. Thereafter, the animals were kept under observation for three hours before determinations of the isolated and perfused hearts.

### 4.3. Determinations of the Isolated and Perfused Hearts

The rats were anesthetized with sodium pentobarbital (60 mg/Kg of body weight) and heparin (1000 U/mL/Kg of body weight). After a thoracotomy, the heart was exposed, and the ascending aorta was referred with the help of a silk thread. The heart was removed, placed in isotonic saline at 4 °C, and connected to the perfusion system through the ascending aorta. The heart was maintained by mechanical activity with Krebs–Henseleit solution (mM)—120 NaCL, 23.4 NaHCO_3_, 4.8 KCL, 1.2 KH_2_PO_4_, 0.86 MgSO_4_, 1.25 CaCL_2_, and 11.0 glucose at pH 7.4 and temperature at 37 °C—through a constant retrograde perfusion (13 mL/min). The perfusion started with an adaptation period of 30 min (5 min with a flow (F) of 25 mL/min and 25 min with F of 13 mL/min). Heart rate (HR) was maintained at 312–324 beats per minute using a Grass stimulator (U7, Grass Instruments Co., Quincy, MA, USA). Coronary flow was regulated with a peristaltic pump (SAD22, Grass Instruments Co., Quincy, MA, USA). Parameters including left intraventricular pressure (LIVP) were recorded by means of a Grass hydropneumatic pressure transducer, to which a catheter with a latex balloon was connected. The balloon was introduced through the mitral valve into the left ventricle and, once inside the cavity, an internal pressure of 5–10 mmHg (diastolic pressure) was applied. After an adaptation period of 30 min, the determinations of coronary vascular resistance (CVR) and cardiac mechanical performance (CMP) were completed. All parameters were recorded using a computer acquisition data system (Grass Poly View). Cardiac mechanical activity (CMA) was calculated as HR × LIVP = CMA [50]. At the end of the experiments, the left ventricle was removed for histological sections and the rest of the heart was homogenized in solution of 25 mM sucrose, 1 mM EDTA, and 10 mM Tris at pH 7.35 with protease inhibitors (1 mM PMSF, 2 μM pepstatin, 2 μM leupeptin, and 0.1% aprotinine. The heart homogenate was centrifuged for 20 min, and at 4 °C, the supernatant was recovered in aliquots and stored at −30 °C and the total proteins were determined by the Bradford method [51].

### 4.4. Histological Preparation

The histological sections of the left ventricle were prepared after the ventricle had been washed in 0.9% NaCL for 30 s and fixed in phosphate buffer (pH 7.4) with formalin at 10% for 24 h. The sections were processed according to conventional histological procedures by Masson’s trichrome stain. Histological sections were analyzed at 25× magnification using a model 63,300 light microscope (Carl Zeiss, Oberkochen, Germany) equipped with a Tucsen (9 megapixels) digital camera and the TSview 7.3.1 software. The cardiac muscle fibers and heart injury zones were analyzed by densitometry using Sigma Scan Pro 5 Image Analysis software (Systat Software Inc., San Jose, CA, USA). The density values are expressed as arbitrary pixel units.

### 4.5. Oxidative Stress Markers: Total Antioxidant Capacity, Glutathione, Selenium Lipid Peroxidation, and Thiols Groups

For all determinations of the OS markers, 100 µg of protein was used, except for selenium (Se) where 200 µg was used. The total antioxidant capacity (TAC) was evaluated according to the method described by Benzie and Strain in the heart homogenate, which was suspended in 1.5 mL buffer composed of 20 mM of Cl_3_FeH_12_O_6_, 300 mM of NaC_2_H_3_O_2_, and 10 mM of 2,4,6-Tris-2-pyridil-s-triazine dissolved in 40 mM of HCL at pH 3.6. The absorbance was measured at 593 nm [52]. The GSH determination was evaluated according to the method described by Rahman et al., at 412 nm in the heart homogenate through the Ellman reactive [53]. The determination of Se was performed according to the method described by Soto et al., in the heart homogenate, and the absorbance was read at 600 nm [54]. Lipid peroxidation (LPO) products were determined in the heart homogenate, making them react with thiobarbituric acid as previously reported and measuring the absorbance at 532 nm [54]. The determination of total thiol groups was performed as previously described by Erel and Neselioglu [55]. Heart homogenate was reduced with 100 µL of KBH_4_, with 10 mM dissolved in CH_3_OH-bidistilled H_2_O (1:1 vol/vol) for 3 min, and then 700 µL of buffer (6.7 mM formaldehyde, 10 mM EDTA, and 100 mM Tris, pH 8.2) was added for 3 min. Finally, 100 µL of DTNB 10 mM in CH_3_OH was added for 4 min. The absorbance was measured at 415 nm.

### 4.6. Determinations of Thioredoxin Reductase (TrxR), Glutathione Peroxidase (GPx), and Glutathione-S-Transferase (GST) Activities

The TrxR activity was indirectly determined by the amount of DTNB in the presence of NADPH to form 2 moles of TNB, according to the method described by Soto et al. [54]. The sample was incubated and monitored at 412 nm for 6 min at 37 °C. The GPx activity was indirectly determined by the amount of oxidized NADPH and was expressed in μmol of NADPH oxidized/min/mg of protein, according to the method previously described, and the sample was incubated and monitored at 340 nm for 6 min at 37 °C [54]. The GST activity was determined according the technique described by Habig et al. The sample was incubated and monitored for 6 min at 37 °C at 340 nm. Values of GST activity were expressed in U/min/mg of protein [56].

### 4.7. Western Blotting for TrxR, GPx, CTH, CBS, iNOS, and eNOS-p

An amount of 50 μg of protein of the heart homogenate was run on 12% SDS-PAGE, blotted onto a polyvinylidene difluoride membrane (0.22 μm Millipore, Billerica, MA, USA), and then blocked for 1 h at room temperature with Tris-buffer solution-0.01% Tween (TBS-T 0.01%) plus 5% non-fat milk. The membranes were incubated overnight at 4 °C with mouse primary monoclonal antibodies, as follows: TrxR (sc-166393), GPx (sc-133160), CTH (sc-374249), CBS (sc-133154), iNOS (sc-7271), and eNOS-p (sc-376751), from Santa Cruz Biotechnology, Santa Cruz, CA, USA, at a final dilution of 1:1000. After that, the membranes were incubated overnight at 4 °C with a secondary antibody that was conjugated with horseradish peroxidase at a dilution of 1:10,000 (Santa Cruz Biotechnology, Santa Cruz, CA, USA). All of the blots were incubated with β-actin antibody as load control. The protein was detected by chemiluminescence assay (Clarity Western ECL Substrate, Bio-Rad Laboratories, Inc., Hercules, CA, USA). Chemiluminescence that was emitted in this process was detected in X-ray films (AGFA, Ortho CP-GU, Agfa HealthCare NV, Belgium). Images from each film were acquired with a GS-800 densitometer (including Quantity One software from Bio-Rad Laboratories, Inc. Hercules, CA, USA). The values of the density of each band are expressed as arbitrary units (AU).

### 4.8. Nitric Oxide Synthase Activity

The NOS activity was quantified using a Clark-type electrode attached to an oximeter (YSI oximeter model 5300A-1). A total of 100 µg of protein from the heart homogenate was incubated at 37 °C for 5 min with 2 mL of buffer containing 50 mM HEPES, 10 units calmodulin, 10 µM BH_4_, 2 mM CaCL_2_, 10 µM L-arginine, 1 mM NADPH, 1 mM FAD, and 1 mM FMN, at pH 7.35. The calibration curve was performed with 0.1 M KI and 0.1 M H_2_SO_4_ in presence of 5 to 160 nM KNO_2_ and values are expressed as a concentration of nitric oxide (NO) nM/mg of protein [57].

### 4.9. Hydrogen Sulfide Concentration

The H_2_S concentration was measured according to the method described by Padiya et al. [58]. A total of 100 µg of protein from the heart homogenate were incubated with 188 µL of 1% zinc acetate, 100 µL distilled water, and 188 µL 20 mM of N,N-dimethyl-phenylene diamine dihydro chloride in 7.2 M of HCL and 150 µL 30 mM of FeCl_3_ in 1.2 M of HCL. The mixture was incubated for 20 min and 376 µL of 10% C_2_HCL_3_O_2_ was added. The sample was centrifuged at 5000 rpm, and the supernatant was measured at a wavelength of 670 nm. The H_2_S concentration was calculated according to the calibration curve of NaHS [59].

### 4.10. Statistical Analysis

The Sigma Plot program 14.5 (SigmaPlot^®^ version 14.5, Systat Software Inc. 2107, San Jose, CA95131 EE.UU, North First Street, Suite 360, Jandel Corporation, San Jose, CA, USA) was used for statistical analysis and graph plotting. The data are presented as mean ± standard error. Statistical significance was determined with Tukey’s one-way ANOVA and post hoc test. A *p* ≤ 0.05 was considered significant.

## 5. Conclusions

The DG treatment probably decreased heart damage caused by LPS through the cross-talk between the H2S and the NO systems, leading to the overexpression or increased activity of GPx, TrxR, and GST, and also favored GSH, Se, and thiols. This favors the enzymatic and non-enzymatic antioxidant systems that comprise the total antioxidant capacity.

## Figures and Tables

**Table 1:** Oxidative stress markers in the rat heart homogenate of the experimental groups.

Variables (mg of Protein)	C	LPS	DG	LPS + DG
TAC (nM)	691.30 ± 64.14	483.91 ± 51.20 *	715.80 ± 52.86	663.54 ± 49.71 *
GSH (µM)	0.009 ± 0.001	0.003 ± 0.0009 **	0.009 ± 0.001	0.010 ± 0.001 **
Se (nM)	0.022 ± 0.001	0.025 ± 0.003	0.022 ± 0.002	0.041 ± 0.001 **
GST activity (GS + DNB µmol)	0.001 ± 0.0001	0.0006 ± 0.00005 *	0.001 ± 0.00007	0.001 ± 0.0001 *
LPO (nmol/MDA)	2.89 ± 0.49	12.86 ± 5.25 **	3.56 ± 0.17	3.78 ± 0.41*
Thiols (µM)	251.74 ± 16.74	166.46 ± 14.35 **	244.451 ± 8.53	272.28 ± 8.39 **

* C and LPS + DG vs. LPS *p* ≤ 0.01. ** C and LPS + DG vs. LPS *p* ≤ 0.001. Abbreviations: TAC = total antioxidant capacity, GSH = glutathione, Se = selenium, GST = glutathione-S-transferase, LPO = lipid peroxidation, C = control, DG = deodorized garlic, LPS = lipopolysaccharide.

## Data Availability

The data in our study are available from the corresponding author upon reasonable request.
